# Association between food-related media content and the eating behaviors of Korean adults according to household type

**DOI:** 10.3389/fnut.2025.1677011

**Published:** 2025-10-08

**Authors:** Ahyoung Yun, Hyein Jung, Byungmi Kim, Yoonjoo Choi

**Affiliations:** 1 Division of Cancer Prevention, National Cancer Control Institute, National Cancer Center, Goyang, Gyeonggi-do, Republic of Korea; 2 Department of Public Health & AI, Graduate School of Cancer Science and Policy, National Cancer Center, Goyang, Gyeonggi-do, Republic of Korea

**Keywords:** Food-related media content, Mukbang, Cookbang, Sulbang, Eating behaviors, Late-night eating, Food delivery/take-out, Dining out

## Abstract

**Background:**

Sociocultural changes and the proliferation of digital platforms have led to the increasing popularity of food-related content in Korea, including Mukbang, Cookbang, and Sulbang. Despite concerns that such content may influence eating habits, research focusing on adults is limited. Therefore, this study examined the association between media content and eating behaviors, stratified by household type, while considering the living environment.

**Methods:**

Data were derived from an online survey conducted by the National Cancer Center, comprising responses from 1,270 participants divided in a 1:1 ratio based on whether they watched food-related content. The participants reported their eating behaviors and viewing habits. Multivariable logistic regression was employed to identify relationships after adjusting for sociodemographic variables, with analyses stratified by household type.

**Results:**

The analysis results showed that in multi-person households, viewing all content types were significantly associated with late-night eating and delivery/take-out meal consumption, with some also related to the frequency of dining out. Conversely, in single-person households, significantly positive associations were exclusively found between Mukbang viewing and late-night eating as well as delivery/take-out meal consumption; Cookbang and Sulbang did not yield statistically significant results. In stratified analysis, more subgroups exhibited associations with eating behaviors, regardless of content type among multi-person households.

**Conclusion:**

The findings suggest that viewing food-related content is linked to dietary behaviors, with effects varying depending on household type. Furthermore, acknowledging the impact of such content on eating behaviors to explore means of utilizing it positively to foster healthy eating habits is imperative.

## Introduction

The consumption of food-related digital content, such as Mukbang (eating broadcasts), has steadily increased and become a global phenomenon (1, 2), In South Korea, Numerous reports indicate that Mukbang is the most popular content type (3), with other formats including Cookbang (cooking broadcasts), and Sulbang (drinking broadcasts) emerging from its popularity (4). The expansion of digital platforms such as YouTube and Instagram (5, 6), the rise in single-person households (7, 8), and the shift toward solitary eating during the coronavirus disease 2019 (COVID-19) pandemic (4) have contributed to heightened interest in and viewership of such content. While food-related content can provide emotional connection and vicarious satisfaction, concerns have been raised that stimulating eating scenes may contribute to excessive appetite and unhealthy dietary behaviors (9, 10).

Concerns regarding food-related content align with changes in dietary behaviors, such as increased dining out, food delivery, and late-night eating in today’s digitalized food culture. In parallel with these media trends, broader lifestyle changes—such as the increase in individuals living alone, greater reliance on app-based food services, and alterations in daily routines owing to COVID-19—have also contributed to notable modifications in eating patterns (11–14). Although food delivery and dining out offer convenience, they often involve foods high in salt, saturated fat, and sugar (12). Moreover, the repeated consumption of delivery meals, late-night eating, and the excessive intake of energy-dense foods have been linked to overeating and an increased risk of chronic diseases (12, 15).

In response to these concerns, several studies have explored the relationship between food-related content and eating behaviors. Some findings indicate that adults who frequently watch Mukbang—based on viewing frequency or duration—are associated with undesirable dietary behaviors (5, 16). Previous studies targeting adolescents have found that watching food-related content associates with an increased risk of unhealthy food consumption, including that of fast food and sugar-sweetened beverages, and more frequent late-night eating and among others (17, 18). Certain studies have investigated both Mukbang and Cookbang in relation to eating behaviors (6, 18), they have often failed to differentiate the content types, despite the distinct characteristics and contexts of each. Moreover, despite the growing popularity of Sulbang, studies examining its association with eating behaviors or alcohol consumption remain exceedingly scarce (4).

Therefore, this study expands on existing research by examining the associations between the viewing of various food-related content types (Mukbang, Cookbang, and Sulbang) and specific dietary behaviors, including late-night eating, food delivery/take-out, and dining out, among Korean adults. Considering that single-person households are more likely to engage in these behaviors (19), the analysis was stratified by household type (single- vs. multi-person households) to reflect the impact of content depending on living environment. Furthermore, subgroup analyses were conducted based on sex, age, and content-viewing characteristics to provide a comprehensive understanding of how food-related content influences eating behaviors. The findings are expected to provide a basis for developing customized strategies for populations with high exposure to specific types of content.

## Methods

### Study participants

This study examined the association between content viewing and the frequency of late-night eating, food delivery/take-out, and dining out among Korean adults, with an emphasis on household type. Data were obtained through an online survey utilizing content-viewing questionnaires developed by the Division of Cancer Prevention, National Cancer Control Institute, National Cancer Center. The survey was administered via an online platform by a professional survey company. The questionnaire encompassed sociodemographic characteristics, content-viewing frequency and patterns, dietary behaviors, and lifestyle factors.

Participants were selected using stratified quota sampling based on age, sex, and region, according to the 2024 Korean population census data. A 1:1 ratio of content viewers (individuals who had watched at least one content type) to non-viewers was maintained. The survey was conducted from July 10 to 23, 2024, until the targeted sample size (*N* = 1,270) was attained. During this process, 418 individuals outside the 20–65-year age range and 461 who did not complete the survey were excluded, and ultimately 1,270 individuals who completed the survey were included.

The study protocol was approved by the Institutional Review Board (IRB) of the National Cancer Center Korea (IRB number: NCC2024-0161). All participants received a written explanation of the study’s purpose, procedures, and data usage, subsequently providing consent to participate before survey commencement.

### General characteristics

The participants’ general characteristics, including age, sex, region, household type, education level, monthly household income, alcohol consumption, physical activity, weight control attempts, perceived health concerns, and obesity status, were obtained through the online survey. These variables were categorized as follows for analysis: age was divided into 20–39- and 40–65-year age groups, while sex was dichotomized into male and female. Region was classified as metropolitan or non-metropolitan. Household type was based on whether participants lived alone (single-person household) or with others (multi-person household). Education level was categorized into “high school or lower” and “college or higher.” Monthly household income was classified into three groups: <3 million Korea won, 3–6 million Korea won, and ≥6 million won. Alcohol consumption was categorized as non-past drinker or current drinker. Physical activity was assessed based on participation in moderate-to-vigorous intensity activity for at least 150 min/week and classified as inactive or active. Weight control attempts were recorded as a binary response (yes or no), while perceived health concerns were classified as low, medium, or high. Obesity status was determined using body mass index (BMI) and categorized into underweight (BMI < 18.5 kg/m^2^), normal weight (18.5 ≤ BMI < 23), overweight (23 ≤ BMI < 25), and obesity (BMI ≥ 25). All categorized variables were utilized to describe the sample characteristics and included as covariates in the regression models.

### Content viewing

Participants were initially queried about their experience with food-related content. Based on their responses, they were classified as viewers or non-viewers and recruited in a 1:1 ratio. Those who reported their experience with food-related content specified the content types they had viewed—Mukbang, Cookbang, and/or Sulbang— selecting multiple options as applicable. Additionally, the number of content types viewed was categorized into 0, 1, 2, or 3. For each content type viewed, participants reported their weekly viewing frequency (< 1 day/week, 1–2 days/week, 3–4 days/week, or 5–6 days/week, daily), average daily viewing duration, preferred video format (short-form, long-form, or both), average number of videos viewed, and the perceived impact of the content on their eating behaviors (no impact, positive, or negative). For analytical purposes, the weekly viewing frequency combined the categories “5–6 days/week” and “daily” categories were combined, and average daily viewing time was classified as <1 h or ≥1 h. In addition, non-viewers and viewers of food content who reported not watching a specific content type (Mukbang, Cookbang, or Sulbang) were also asked to indicate their reasons for non-viewing along with their perception of the impact of that content on eating behaviors, using the same response categories (no impact, positive, or negative).

### Eating behaviors

Regarding eating behaviors, participants reported the frequency (per week) of three specific behaviors: late-night eating, delivery/take-out meal intake, and dining out. For analytical purposes, response values were dichotomized according to the median for each behavior. Specifically, consuming late-night snacks or delivery/take-out meals two or more times per week and dining out three or more times per week were established as cutoff points to define comparison groups.

### Statistical analysis

All analyses were stratified by household type (single- vs. multi-person households). To examine group differences between household types, chi-square tests and independent t-tests were employed for categorical and continuous variables, respectively. Within each household group, multivariable logistic regression analyses were conducted by content type (Mukbang, Cookbang, and Sulbang) to assess the associations between content-viewing behaviors and dietary outcomes. Odds ratios (ORs) with 95% confidence intervals (CIs) were calculated, adjusting for covariates such as age, sex, region, education level, monthly household income, alcohol consumption, physical activity, weight control attempts, perceived health concerns, and obesity status. Statistical significance was set at *p* < 0.05, and all statistical analyses were performed using SAS (version 9.4; SAS Institute Inc., Cary, NC, United States).

## Results

### Participants’ general characteristics

All results were stratified by household type (single- vs. multi-person households), as presented in Table 1, which outlines the participants’ general characteristics by household type. Of the 1,270 participants, 224 (17.6%) and 1,046 (82.4%) belonged to single- and multi-person households, respectively. Among the participants, 38.2% were aged 20–39 years, and 61.8% were aged 30–65 years; 51.5% were male, and 48.5% were female. Single-person households exhibited a higher proportion of younger adults and a lower proportion of individuals with a monthly household income than multi-person households (*p* < 0.0001).

**Table 1:** General characteristics.

Variable	Total	Single	Multi	*p*-value
Total	1,270 (100.0)	224 (17.6)	1,046 (82.4)	
Age				
20–39 years	485 (38.2)	129 (57.6)	356 (34.0)	<0.0001
40–65 years	785 (61.8)	95 (42.4)	690 (66.0)	
Sex				
Male	654 (51.5)	122 (54.5)	532 (50.9)	0.327
Female	616 (48.5)	102 (45.5)	514 (49.1)	
Region				
Metropolitan area	669 (52.7)	115 (51.3)	554 (53.0)	0.659
Non-metropolitan area	601 (47.3)	109 (48.7)	492 (47.0)	
Education level				
High school or lower	231 (18.2)	35 (15.6)	196 (18.7)	0.273
College or higher	1,039 (81.8)	189 (84.4)	850 (82.3)	
Monthly household income				
<3 million won	317 (25.0)	129 (57.6)	188 (18.0)	<0.0001
3–6 million won	577 (45.4)	87 (38.9)	490 (46.8)	
≥6 million won	376 (29.6)	8 (3.6)	368 (35.2)	
Alcohol consumption				
Non-past drinker	387 (30.5)	76 (33.9)	311 (29.7)	0.216
Current drinker	883 (69.5)	148 (66.1)	735 (70.3)	
Physical activity				
Inactive	1,154 (90.9)	211 (94.2)	943 (90.2)	0.057
Active	116 (9.1)	13 (5.8)	103 (9.8)	
Weight control attempts				
No	597 (47.0)	113 (50.4)	484 (46.3)	0.256
Yes	673 (53.0)	111 (49.6)	562 (53.7)	
Health concerns				
Low	138 (10.9)	29 (12.9)	109 (10.4)	0.144
Medium	520 (40.9)	100 (44.6)	420 (40.2)	
High	612 (48.2)	95 (42.4)	517 (49.4)	
Obesity				
Underweight	86 (6.8)	15 (6.7)	71 (6.8)	0.076
Normal weight	531 (44.2)	116 (51.8)	445 (42.5)	
Overweight	295 (23.2)	46 (20.5)	249 (23.8)	
Obese	328 (25.8)	47 (21.0)	281 (26.9)	

Values are presented as *N* (%).

*p*-values were calculated using the chi-square test for categorical variables.

Physical activity was defined as moderate-to-vigorous intensity activity for ≥150 min/week.

BMI, body mass index. Obesity status: underweight: BMI <18.5 kg/m^2^; Normal, 18.5 ≤ BMI <23 kg/m^2^; Overweight, 23 ≤ BMI <25 kg/m^2^; Obese, BMI ≥25 kg/m^2^.

### Participants’ content viewing and eating behaviors

Table 2 illustrates the participants’ content-viewing statuses, content types, and eating behaviors by household type. Content viewers and non-viewers were included in a 1:1 ratio. Among the viewers, 90.6% (single: 89.2%; multi: 90.8%) reported watching Mukbang, 66.1% (single: 89.2%; multi: 90.8%) watched Cookbang, and 40.3% (single: 42.3%; multi: 39.9%) viewed Sulbang. The frequency of eating behaviors was approximately 1.16 times per week for late-night eating (single: 1.16 times; multi: 1.17 times), 1.34 times for delivery or take-out meals (single 1.35 times; multi 1.34 times), and 2.5 times for dining out (single: 2.8 times; multi: 2.41 times). The frequency of dining out was significantly higher in single-person households (*p* < 0.05).

**Table 2:** Content viewing and eating behaviors by household type.

Variable	Total	Single	Multi	*p*-value
Total	1,270 (100.0)	224 (17.6)	1,046 (82.4)	
Content viewing				
No	635 (50.0)	113 (50.4)	522 (49.9)	0.883
Yes	635 (50.0)	111 (49.6)	524 (50.1)	
Content				
Mukbang	575 (90.6)	99 (89.2)	476 (90.8)	
Cookbang	420 (66.1)	73 (65.8)	347 (66.2)	
Sulbang	256 (40.3)	47 (42.3)	209 (39.9)	
Dietary behaviors				
Late-night eating	1.16 ± 1.46	1.16 ± 1.32	1.17 ± 1.48	0.965
Once a week or less	867 (68.3)	151 (67.4)	716 (68.5)	0.761
Twice per week or more	403 (31.7)	73 (32.6)	350 (31.5)	
Delivery or take-out	1.34 ± 1.50	1.35 ± 1.47	1.34 ± 1.25	0.897
Once a week or less	831 (65.4)	140 (62.5)	691 (66.1)	0.309
Twice per week or more	439 (34.6)	84 (37.5)	355 (33.9)	
Dining out	2.48 ± 2.36	2.80 ± 2.75	2.41 ± 2.27	0.026
Twice per week or less	796 (62.7)	130 (58.0)	666 (63.7)	0.114
Thrice per week or more	474 (37.3)	94 (42.0)	380 (36.3)	

Values are presented as the mean ± SD or *N* (%).

*p*-values were calculated using the independent *t*-test and chi-square test for continuous and categorical variables, respectively.

Supplementary Table 1 presents the participants’ content-viewing characteristics by content type, encompassing viewing frequency, viewing duration, preferred video format, number of videos, and the perceived impact of content on eating behaviors. For Mukbang, no significant differences were observed between the household types. In contrast, a significantly greater proportion of participants in multi-person households reported that Cookbang positively influenced their eating behavior compared to single multi-person households (*p* < 0.001). Additionally, single-person households had a higher percentage of participants who watched Sulbang for over an hour than multi-person households (*p* < 0.05). No other significant differences were identified between the household types.

The responses of non-viewers for each content type are summarized in Supplementary Table 2. Regarding Mukbang, the predominant reason for non-viewing cited by both single- and multi-person households was that it was perceived as a “waste of time” (single: 40.8%; multi: 44.9%), with significant differences noted between the two groups (*p* < 0.05). In particular, 54% of participants in single-person households believed that Mukbang could negatively impact their eating behaviors, a proportion that was significantly higher than that in multi-person households (*p* < 0.05). In contrast, no significant differences were noted between the household types regarding Cookbang and Sulbang.

### ORs for eating behaviors according to content-viewing status by household type

Table 3 illustrates the association between food-related content—specifically Mukbang, Cookbang, and Sulbang—and dietary behaviors, stratified by household type. In both single- and multi-person households, participants who viewed Mukbang exhibited greater odds of engaging in late-night eating (single: OR = 2.193, 95% CI: 1.169–4.115; multi: OR = 1.731, 95% CI: 1.314–2.279) and delivery/take-out meal consumption (single: OR = 1.912, 95% CI: 1.008–3.625; multi: OR = 2.360, 95% CI: 1.784–3.121) than non-viewers. Among multi-person households, Cookbang viewing showed higher odds of late-night eating (OR = 2.381, 95% CI: 1.780–3.185), food delivery/take-out (OR = 2.383, 95% CI: 1.782–3.187), and dining out (OR = 1.505, 95% CI: 1.133–1.997), while Sulbang viewing was associated with higher odds of late-night eating (OR = 2.692, 95% CI: 1.942–3.733) and food delivery/take-out (OR = 2.897, 95% CI: 2.077–4.040). No significant associations were identified for Cookbang or Sulbang viewing among single-person households.

**Table 3:** Associations between content viewing and eating behaviors by household type.

Variable	Single			Multi		
	Late-night eating	Delivery or take-out	Dining out	Late-night eating	Delivery or take-out	Dining out
Mukbang viewing						
No	1.000 (reference)	1.000 (reference)	1.000 (reference)	1.000 (reference)	1.000 (reference)	1.000 (reference)
Yes	2.193 (1.169–4.115)	1.912 (1.008–3.625)	1.488 (0.791–2.800)	1.731 (1.314–2.279)	2.36 (1.784–3.121)	1.234 (0.942–1.616)
Cookbang viewing						
No	1.000 (reference)	1.000 (reference)	1.000 (reference)	1.000 (reference)	1.000 (reference)	1.000 (reference)
Yes	1.251 (0.647–2.419)	1.271 (0.645–2.504)	0.980 (0.498–1.929)	2.381 (1.780–3.185)	2.383 (1.782–3.187)	1.505 (1.133–1.997)
Sulbang viewing						
No	1.000 (reference)	1.000 (reference)	1.000 (reference)	1.000 (reference)	1.000 (reference)	1.000 (reference)
Yes	1.535 (0.735–3.207)	0.903 (0.417–1.954)	0.940 (0.437–2.019)	2.692 (1.942–3.733)	2.897 (2.077–4.040)	1.183 (0.852–1.642)

Values are expressed as odds ratios (95% confidence intervals).

Odds ratios were adjusted for age, sex, region, educational level, monthly household income, household type, alcohol consumption, physical activity, weight control attempts, health concern level, and obesity.

Stratified analyses by sex, age, and region within each household type are presented in Table 4. In single-person households, Mukbang viewing was significantly associated with late-night eating among participants aged 20–39 years and those residing in metropolitan areas (20–39 years: OR = 2.470, 95% CI: 1.067–5.717; metropolitan areas: OR = 5.107, 95% CI: 1.904–13.703). Additionally, it was linked to food delivery/take-out among women (OR = 4.596, 95% CI: 1.428–14.798). Conversely, Cookbang viewing was associated with lower odds of dining out among participants aged 40–65 years (OR = 0.173, 95% CI: 0.036–0.842) and higher odds of food delivery/take-out among women (OR = 3.490, 95% CI: 1.134–10.747). Sulbang viewing was associated with higher odds of late-night eating and food delivery/take-out among men (late-night eating: OR = 2.523, 95% CI: 1.459–4.362; food delivery/take-out: OR = 2.278, 95% CI: 1.315–3.947), as well as greater odds of all three behaviors among women (late-night eating: OR = 2.967, 95% CI: 1.958–4.497; food delivery/take-out: OR = 3.554, 95% CI: 2.324–5.433; dining out: OR = 1.639, 95% CI: 1.077–2.493). In contrast, within multi-person households, most subgroups demonstrated significant associations across content types and dietary behaviors. In addition, the association between the number of content types viewed and dietary behaviors are presented in Supplementary Table 3.

**Table 4:** Stratified associations between content viewing and eating behaviors.

Variable	No	Mukbang			Cookbang			Sulbang		
		Viewing			Viewing			Viewing		
		Late night eating	Delivery or take-out	Dining out	Late night eating	Delivery or take-out	Dining out	Late night eating	Delivery or take-out	Dining out
Single										
Age										
20–39 years	1.000 (reference)	2.470 (1.067–5.717)	1.713 (0.748–3.926)	1.872 (0.822–4.266)	1.525 (0.683–3.406)	0.854 (0.379–1.924)	1.824 (0.812–4.100)	1.847 (0.762–4.479)	0.774 (0.305–1.964)	1.302 (0.528–3.210)
40–65 years	1.000 (reference)	2.446 (0.810–7.389)	2.806 (0.792–9.939)	1.453 (0.449–4.706)	0.668 (0.157–2.842)	3.388 (0.680–16.879)	0.173 (0.036–0.842)	1.368 (0.241–7.762)	2.754 (0.488–15.547)	1.083 (0.212–5.532)
Sex										
Male	1.000 (reference)	2.245 (0.923–5.459)	1.207 (0.517–2.819)	1.725 (0.737–4.037)	1.119 (0.406–3.083)	0.617 (0.227–1.677)	1.399 (0.520–3.761)	2.523 (1.459–4.362)	2.278 (1.315–3.947)	0.773 (0.441–1.354)
Female	1.000 (reference)	2.737 (0.942–7.952)	4.596 (1.428–14.798)	1.149 (0.397–3.325)	1.472 (0.524–4.132)	3.490 (1.134–10.747)	0.689 (0.234–2.025)	2.967 (1.958–4.497)	3.554 (2.324–5.433)	1.639 (1.077–2.493)
Region										
Metropolitan area	1.000 (reference)	5.107 (1.904–13.703)	2.604 (0.982–6.903)	1.452 (0.556–3.796)	2.089 (0.771–5.662)	1.556 (0.548–4.420)	1.896 (0.659–5.453)	2.194 (0.777–6.196)	0.819 (0.279–2.410)	0.948 (0.308–2.911)
Non-metropolitan area	1.000 (reference)	0.804 (0.304–2.128)	1.595 (0.572–4.448)	1.745 (0.674–4.521)	0.759 (0.275–2.096)	0.940 (0.339–2.610)	0.504 (0.183–1.383)	0.656 (0.204–2.106)	0.724 (0.209–2.516)	0.807 (0.254–2.561)
Multi										
Age										
20–39 years	1.000 (reference)	2.171 (1.341–3.514)	2.148 (1.352–3.412)	0.787 (0.486–1.274)	2.159 (1.305–3.571)	1.975 (1.210–3.224)	1.292 (0.783–2.133)	2.993 (1.010–8.868)	0.442 (0.141–1.384)	0.792 (0.269–2.334)
40–65 years	1.000 (reference)	1.563 (1.108–2.204)	2.520 (1.755–3.619)	1.489 (1.062–2.087)	2.635 (1.830–3.795)	2.689 (1.858–3.891)	1.656 (1.160–2.364)	0.840 (0.256–2.759)	2.534 (0.693–9.261)	1.740 (0.482–6.288)
Sex										
Male	1.000 (reference)	1.984 (1.364–2.886)	2.801 (1.880–4.175)	1.469 (1.010–2.137)	2.906 (1.952–4.325)	2.566 (1.710–3.852)	1.937 (1.299–2.888)	3.400 (2.202–5.248)	3.469 (2.232–5.390)	1.232 (0.798–1.903)
Female	1.000 (reference)	1.465 (0.963–2.229)	1.931 (1.288–2.893)	1.015 (0.676–1.524)	1.900 (1.218–2.962)	2.291 (1.488–3.528)	1.271 (0.830–1.946)	1.974 (1.171–3.327)	2.296 (1.366–3.858)	1.294 (0.770–2.175)
Region										
Metropolitan area	1.000 (reference)	1.604 (1.101–2.336)	2.161 (1.473–3.170)	1.144 (0.795–1.647)	2.290 (1.545–3.396)	2.030 (1.370–3.009)	1.131 (0.773–1.657)	2.896 (1.846–4.545)	2.623 (1.665–4.132)	0.917 (0.587–1.434)
Non-metropolitan area	1.000 (reference)	1.945 (1.284–2.946)	2.658 (1.747–4.044)	1.461 (0.966–2.209)	2.622 (1.682–4.087)	3.158 (2.017–4.945)	2.272 (1.469–3.513)	2.547 (1.565–4.147)	3.376 (2.040–5.585)	1.654 (1.013–2.701)

Values are expressed as odds ratios (95% confidence intervals).

Odds ratios were adjusted for age, sex, region, educational level, monthly household income, household type, alcohol consumption, physical activity, weight control attempts, health concern level, and obesity.

### ORs for eating behaviors according to content-viewing characteristics in multi-person households

**Figure 1:**
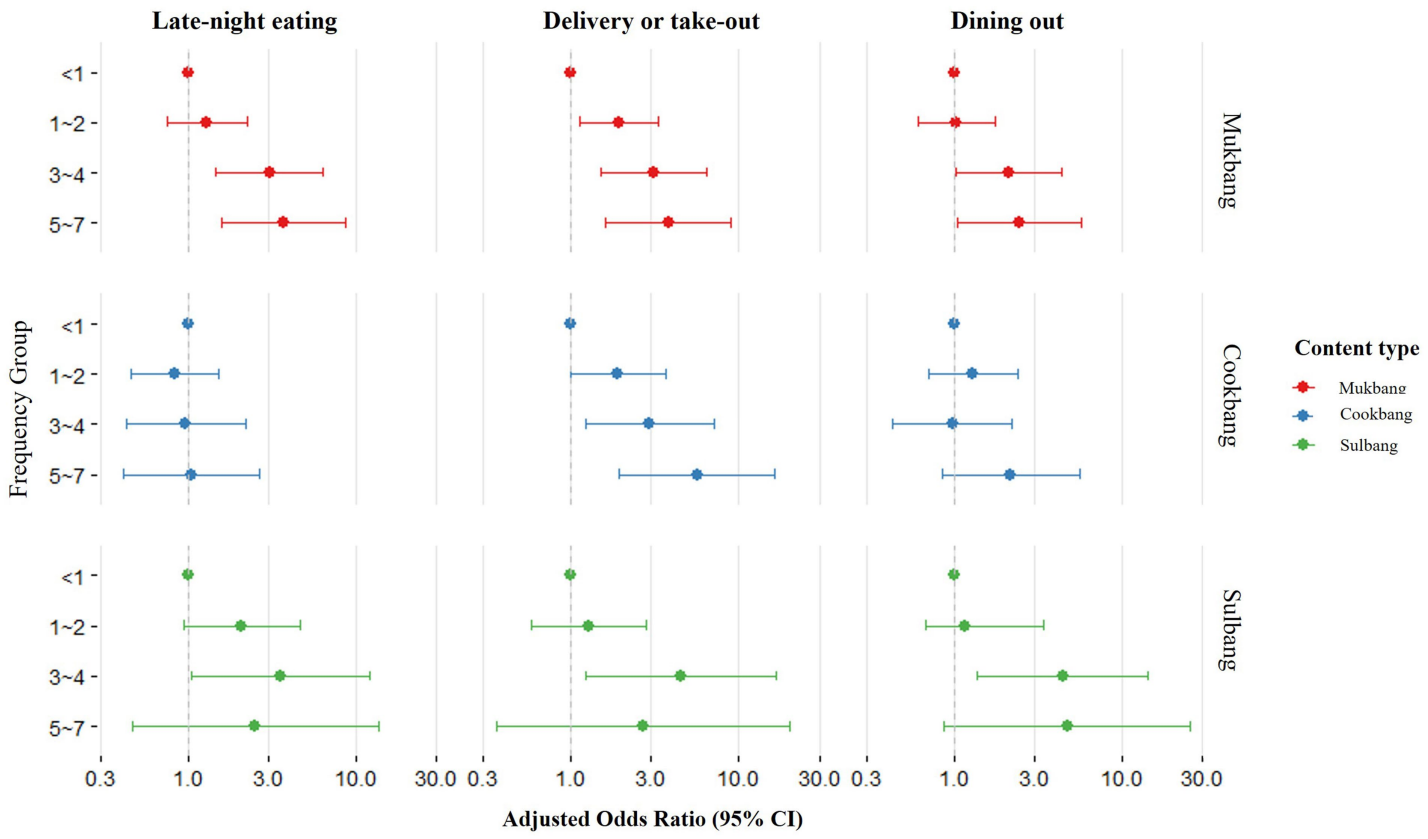
Adjusted odds ratios for eating behaviors by viewing frequency and content type in multi-person households. Odds ratios were adjusted for age, sex, region, educational level, monthly household income, household type, alcohol consumption, physical activity, weight control attempts, health concern level, obesity, and viewing characteristics.

Figure 1 illustrates the odds of dietary behaviors based on content-specific viewing characteristics within multi-person households. For Mukbang, a higher viewing frequency was associated with higher odds of late-night eating, food delivery/take-out, and dining out. In the case of Cookbang, more frequent viewing was linked to a higher likelihood of food delivery/take-out. Participants who reported watching Sulbang 3–4 times per week exhibited elevated odds for all three behaviors: late-night eating, food delivery/take-out, and dining out. Detailed regression results are provided in Supplementary Table 4.

## Discussion

This study examined the relationship between food-related digital content and eating behaviors, specifically late-night eating, food delivery/take-out, and dining out, among Korean adults, stratified by household type. The results indicated that content viewing was significantly associated with dietary behaviors, with variations observed by household type and viewing characteristics. Notably, among multi-person households, viewing all content types exhibited significant associations with higher odds of late-night eating and food delivery/take-out, and in certain instances, with dining out. In contrast, among single-person households, exclusive Mukbang viewing was positively associated with higher odds of late-night eating and delivery/take-out behaviors, while Cookbang and Sulbang did not yield statistically significant results.

Previous studies indicate that single-person households are more likely to engage with food-related content (7, 20) and to engage in eating patterns such as delivery/take-out meal intake and dining out than multi-person households (19). Nevertheless, this study found no significant differences between single- and multi-person households concerning the content-viewing statuses, and the frequency of late-night eating or food delivery/take-out; only the frequency of dining out was significantly higher among single-person households. This discrepancy may be attributable to the stratified nature of the sample, which was based on viewing status, age, and sex. Additionally, these results may be due to the insufficient consideration of age-related differences in dietary patterns and content viewing rates.

Stratified analysis showed that single-person households exhibited limited associations, likely because convenience-oriented eating patterns (e.g., dining out, delivery) were already established (19, 21). In contrast, multi-person households showed consistent associations across content types, reflecting the stronger influence of social interactions on food choices (22, 23).

Mukbang frequently features excessive food intake, often involving late-night eating and the consumption of energy-dense foods that are salty, sweet, or greasy (24). Such content may stimulate viewers’ appetites, justify overeating behaviors (25), and contribute to unhealthy eating habits and potential health problems (26). This phenomenon may contribute to the social norms for dietary behavior, which may be a potential concern from a public health perspective. Consistent with these concerns, this study found significant associations between Mukbang viewing and behaviors such as late-night eating and the use of delivery or take-out services, corroborating the notion of these potential risks. Similarly, Cookbang and Sulbang showed positive association with the high frequency of eating behaviors. By accompanying food, these contents can stimulate viewers’ appetites and prompt late-night meals. Furthermore, this may be attributed to interaction effects that obscure the independent influence of each content type owing to the combined viewing of multiple content types. Nonetheless, the present study identified a lower association between Cookbang viewing and the dining out among single-person households aged 40–65 years. This result is consistent with previous study reporting that Cookbang increased desire and practice to cook (9). Likewise, expanding food-related contents including health food information and cooking guidance may represent a beneficial strategy to reduce unhealthy eating behaviors.

In this study, content viewing was weakly associated with dining out than with late-night eating or food delivery/take-out. These findings may be linked to the sociocultural changes resulting from the COVID-19 pandemic, which led to a reduction in traditional dining opportunities and a considerable increase in the use of food delivery services (14, 15). In addition, numerous food-related programs are broadcast late at night (after dinner), potentially heightening nighttime cravings (24). Since food delivery services are frequently utilized after dinner, particularly during late-night hours, late-night eating and food delivery behaviors are closely linked in practice (15, 27). Consequently, exposure to such content may be linked with increased likelihood of engaging in both behaviors. Delivery and dining-out meals are often high in calories, fat, and sodium, potentially contributing to unhealthy eating habits and elevating the risk of obesity and other chronic diseases (27, 28). In addition, late-night eating is associated with unhealthy food intake, disproportionate energy consumption, and excessive sodium intake, which can cause physical health problems, such as obesity, gastrointestinal issues, and poor sleep quality (19, 29, 30). These characteristics indicate that food-related content may encourage unhealthy eating behaviors, offering valuable insights into the mechanisms underlying these associations.

To understand this cultural phenomenon and support a shift toward a healthier eating environment, management of exposure to food-related media including viewing-time management and late-night exposure restrictions should be considered. In addition, considering the growth of food-delivery environment, delivery platform-based interventions such as adding menu labeling (31), offering low salt/sugar options (11), could be beneficial. Furthermore, as associations may differ depending on household types, family-based approaches such as programs that encourage home-prepared meals should target multi-person households as well as single-person households.

This study has certain limitations. First, because of its cross-sectional nature, it could not ascertain causality or influence, merely identifying associations between content viewing and dietary behaviors. For instance, whether individuals watch Mukbang more frequently because they often consume delivery food or whether watching Mukbang leads to increased food delivery behavior remains unclear. Second, all variables were self-reported, complicating the assessment of the actual frequency and extent of content viewing, dietary behaviors or covariates. These self-reported data may have response biases, including misunderstanding of the items and social-desirability bias to look good (32). For instance, while the survey item inquired about the number of meals eaten outside the home per week, some participants might have interpreted this in terms of the number of days, potentially leading to underestimation. Third, selection bias may have occurred due to the use of stratified quota sampling based on sex, age, region, and content viewing status (33). Fourth, although dichotomizing continuous variables may facilitate interpretation, it results in a loss of information and power, and can introduce bias depending on the choice of cutpoint (34). Finally, due to overlapping viewing patterns, many participants reported watching multiple types of food-related content, making it difficult to isolate the independent effects of Mukbang, Cookbang, and Sulbang on dietary behaviors. Additional analyses considering the number of content types showed that a greater variety of content exposure was high associated with eating behaviors in multi-person households. However, it remains unclear how the different content types may interact to produce these effects. Future research should address these limitations by examining and incorporating contextual factors related to content consumption, such as viewing time (e.g., late at night) and actual dietary intake.

Despite these limitations, this study possesses several strengths. First, unlike previous research, it simultaneously analyzed multiple food-related content types: Mukbang, Cookbang, and Sulbang, considering sociodemographic characteristics. Second, the study provided a more nuanced understanding of the associations between content viewing and eating behaviors by stratifying the analysis according to household type (single- vs. multi-person households) and viewing characteristics (e.g., frequency). The findings, particularly in multi-person households, reveal significant associations between various content types and unhealthy eating behaviors, thereby offering empirical support for the potential behavioral impact of these media.

In conclusion, this study investigated the association between food-related content and eating behaviors, focusing on variations across household types. The findings indicate that content viewing is linked to behaviors such as late-night eating, food delivery/take-out, and dining out, with these associations being more pronounced in multi-person households. These results underscore the imperativeness of considering both the media environment and household context in understanding dietary behaviors. In addition, acknowledging the influence of content highlights the necessity of developing strategies that promote healthy eating habits and leverage food-related media for educational purposes.

## Data Availability

The datasets presented in this article are not readily available because this study utilized internal survey data collected by the National Cancer Center, which are not available for external access and are exclusively restricted to authorized personnel. Requests to access the datasets should be directed to Ahyoung Yun, yay1210@ncc.re.kr.
