# Correction: Association between food-related media content and the eating behaviors of Korean adults according to household type

**DOI:** 10.3389/fnut.2025.1754434

**Published:** 2025-12-11

**Authors:** Ahyoung Yun, Hyein Jung, Byungmi Kim, Yoonjoo Choi

**Affiliations:** 1 Division of Cancer Prevention, National Cancer Control Institute, National Cancer Center, Goyang, Gyeonggi-do, Republic of Korea; 2 Department of Public Health & AI, Graduate School of Cancer Science and Policy, National Cancer Center, Goyang, Gyeonggi-do, Republic of Korea

**Keywords:** food-related media content, Mukbang, Cookbang, Sulbang, Eating behaviors, Late-night eating, Food delivery/take-out, Dining out

In the published article, the incorrect Funding statement and grant number were provided. The incorrect grant number was (NCC-2311450-3). The correct grant numbers are (NCC-2311450-3 and NCC-2511621-1). The correct **Funding** Statement appears below.

The author(s) declared that financial support was received for this work and/or its publication. This research was supported by the National Cancer Center (NCC), Republic of Korea research grant (NCC-2311450-3 and NCC-2511621-1).

The original version of this article has been updated.

