# Supplementary material for: Association between food-related media content and the eating behaviors of Korean adults according to household type
Source: Front Nutr. 2025 Oct 8;12:1677011. doi: 10.3389/fnut.2025.1677011 (PMC12540150; doi:10.3389/fnut.2025.1677011)
Supplement: Supplementary file 1 [file Table_1.DOCX]

Supplementary Table 1. Content viewing characteristics by household type

|  | Mukbang | | | Cookbang | | | Sulbang | | |
| --- | --- | --- | --- | --- | --- | --- | --- | --- | --- |
|  | Single | Multi |  | Single | Multi |  | Single | Multi |  |
|  | n=99 | n=476 | p-value | n=73 | n=347 | p-value | n=47 | n=209 | p-value |
| **Frequency** |  |  |  |  |  |  |  |  |  |
| <1 days/week | 35 (35.4) | 167 (35.1) | 0.904 | 18 (24.7) | 93 (26.8) | 0.155 | 20 (42.6) | 96 (45.9) | 0.077 |
| 1–2 days/week | 36 (36.4) | 175 (36.8) |  | 38 (52.1) | 150 (43.2) |  | 24 (51.1) | 72 (34.4) |  |
| 3–4 days/week | 17 (17.1) | 71 (14.9) |  | 14 (19.2) | 60 (17.3) |  | 2 (4.3) | 28 (13.4) |  |
| 5–7 days/week | 11 (11.1) | 63 (13.2) |  | 3 (4.1) | 44 (12.7) |  | 1 (2.1) | 13 (6.2) |  |
| **Time** |  |  |  |  |  |  |  |  |  |
| <1 h/day | 59 (59.6) | 287 (60.3) | 0.897 | 49 (67.1) | 206 (59.4) | 0.217 | 17 (36.2) | 121 (57.9) | 0.007 |
| ≥1 h/day | 40 (40.4) | 180 (39.7) |  | 24 (32.9) | 141 (40.6) |  | 30 (63.8) | 88 (42.1) |  |
| **Length** |  |  |  |  |  |  |  |  |  |
| Short-form | 30 (30.3) | 137 (28.8) | 0.241 | 15 (20.5) | 92 (26.5) | 0.393 | 8 (17.0) | 36 (17.2) | 0.555 |
| Long-form | 46 (46.5) | 189 (39.7) |  | 34 (46.6) | 134 (38.6) |  | 29 (61.7) | 113 (54.1) |  |
| Both | 23 (23.2) | 150 (31.5) |  | 24 (32.9) | 121 (34.9) |  | 10 (21.3) | 60 (28.7) |  |
| **Number** |  |  |  |  |  |  |  |  |  |
| 1 | 36 (36.4) | 195 (41.0) | 0.059 | 36 (49.3) | 154 (44.4) | 0.702 | 33 (70.2) | 123 (58.9) | 0.310 |
| 2 | 35 (35.4) | 114 (23.9) |  | 14 (19.2) | 79 (22.8) |  | 7 (14.9) | 50 (23.9) |  |
| 3 or more | 28 (28.3) | 167 (35.1) |  | 23 (31.5) | 114 (32.9) |  | 7 (14.9) | 36 (17.2) |  |
| **Influence on dietary behaviors** |  |  |  |  |  |  |  |  |  |
| No | 46 (46.5) | 186 (39.1) | 0.310 | 29 (39.7) | 68 (19.6) | <0.001 | 25 (53.2) | 93 (44.5) | 0.498 |
| Positive | 27 (27.3) | 132 (27.7) |  | 42 (57.5) | 252 (72.6) |  | 6 (12.8) | 38 (18.2) |  |
| Negative | 26 (26.3) | 158 (33.2) |  | 2 (2.7) | 27 (7.8) |  | 16 (34.0) | 78 (37.3) |  |

Values are presented as N (%).

P-values were obtained using chi-squared tests for categorical variables.
