# Supplementary material for: Association between food-related media content and the eating behaviors of Korean adults according to household type
Source: Front Nutr. 2025 Oct 8;12:1677011. doi: 10.3389/fnut.2025.1677011 (PMC12540150; doi:10.3389/fnut.2025.1677011)
Supplement: Supplementary file 2 [file Table_2.DOCX]

Supplementary Table 2. Household type-based reasons for not watching and perceived behavioral influence by content type

|  | Mukbang | | | | Cookbang | | | Sulbang | | |
| --- | --- | --- | --- | --- | --- | --- | --- | --- | --- | --- |
|  | Single (n=125) | Multi (n=570) | p-value | Single (n=151) | | Multi (n=699) | p-value | Single (n=177) | Multi (n=837) | p-value |
| **Reasons for not watching** |  |  |  |  | |  |  |  |  |  |
| To avoid cravings for food or alcohol | 6 (4.8) | 56 (9.8) | 0.027 | 13 (8.6) | | 68 (9.7) | 0.132 | 7 (4.0) | 56 (6.7) | 0.736 |
| To adhere to a diet | 3 (2.4) | 7 (1.2) |  | 8 (5.3) | | 20 (2.9) |  | 4 (2.3) | 18 (2.2) |  |
| To watch other preferred content | 18 (14.4) | 55 (9.6) |  | 25 (16.6) | | 133 (19.0) |  | 34 (19.2) | 129 (15.4) |  |
| Difficulty stopping once started | 4 (3.2) | 17 (3.0) |  | 8 (5.3) | | 46 (6.6) |  | 7 (4.0) | 30 (3.6) |  |
| Lack of interest in content | 15 (12.0) | 91 (16.0) |  | 37 (24.5) | | 109 (15.6) |  | 41 (23.2) | 218 (26.0) |  |
| To perceive as a waste of time | 51 (40.8) | 256 (44.9) |  | 32 (21.2) | | 172 (24.6) |  | 50 (28.2) | 242 (28.9) |  |
| Lack of time | 0 (0.0) | 12 (2.1) |  | 4 (2.6) | | 36 (5.2) |  | 4 (2.3) | 23 (2.7) |  |
| Low video-watching habits in general | 28 (22.4) | 76 (13.3) |  | 24 (15.9) | | 115 (16.5) |  | 30 (16.9) | 121 (14.5) |  |
| **Influence on dietary or drinking behaviors** |  |  |  |  | |  |  |  |  |  |
| No | 9 (7.2) | 40 (7.0) | 0.013 | 35 (23.2) | | 192 (27.5) | 0.553 | 4 (2.3) | 31 (3.7) | 0.115 |
| Positive | 48 (38.4) | 299 (52.5) |  | 33 (21.9) | | 141 (20.2) |  | 99 (55.9) | 521 (62.2) |  |
| Negative | 68 (54.4) | 231 (40.5) |  | 83 (55.0) | | 366 (52.4) |  | 74 (41.8) | 285 (34.1) |  |

Values are presented as N (%).

P-values were obtained using chi-squared tests for categorical variables.
