# Supplementary material for: Association between food-related media content and the eating behaviors of Korean adults according to household type
Source: Front Nutr. 2025 Oct 8;12:1677011. doi: 10.3389/fnut.2025.1677011 (PMC12540150; doi:10.3389/fnut.2025.1677011)
Supplement: Supplementary file 3 [file Table_3.DOCX]

|  | Single | | | Multi | | |
| --- | --- | --- | --- | --- | --- | --- |
|  | Late-night eating | Delivery or take-out | Dining out | Late-night eating | Delivery or take-out | Dining out |
| **Number of content types** |  |  |  |  |  |  |
| 0 | 1.000 (reference) | 1.000 (reference) | 1.000 (reference) | 1.000 (reference) | 1.000 (reference) | 1.000 (reference) |
| 1 | 1.24 (0.547–2.812) | 1.275 (0.553–2.94) | 1.334 (0.602–2.959) | 1.144 (0.774–1.691) | 1.305 (0.886–1.925) | 1.055 (0.73–1.527) |
| 2 | 2.329 (0.975–5.564) | 2.712 (1.093–6.729) | 1.289 (0.522–3.182) | 1.969 (1.326–2.924) | 2.031 (1.364–3.023) | 1.193 (0.807–1.764) |
| 3 | 1.842 (0.77–4.407) | 1.165 (0.472–2.877) | 1.14 (0.464–2.8) | 3.152 (2.161–4.598) | 4.08 (2.769–6.011) | 1.522 (1.05–2.206) |

Supplementary Table 3. Associations between number of content types viewed and eating behaviors
