# Supplementary material for: Association between food-related media content and the eating behaviors of Korean adults according to household type
Source: Front Nutr. 2025 Oct 8;12:1677011. doi: 10.3389/fnut.2025.1677011 (PMC12540150; doi:10.3389/fnut.2025.1677011)
Supplement: Supplementary file 4 [file Table_4.DOCX]

Supplementary Table 4. Associations between content viewing characteristics and eating behaviors in muti-person households

|  | Mukbang | | | Cookbang | | | Sulbang | | |
| --- | --- | --- | --- | --- | --- | --- | --- | --- | --- |
|  | Late-night eating | Delivery or take-out | Dining out | Late-night eating | Delivery or take-out | Dining out | Late-night eating | Delivery or take-out | Dining out |
| **Frequency** |  |  |  |  |  |  |  |  |  |
| <1 day/week | 1.000 (reference) | 1.000 (reference) | 1.000 (reference) | 1.000 (reference) | 1.000 (reference) | 1.000 (reference) | 1.000 (reference) | 1.000 (reference) | 1.000 (reference) |
| 1–2 days/week | 1.297  (0.745–2.26) | 1.940  (1.137–3.311) | 1.033  (0.607–1.756) | 0.838  (0.459–1.531) | 1.903  (0.997–3.633) | 1.302  (0.713–2.377) | 2.094  (0.936–4.684) | 1.285  (0.585–2.821) | 1.517  (0.676–3.402) |
| 3–4 days/week | 3.044  (1.469–6.31) | 3.105  (1.510–6.384) | 2.108  (1.025–4.334) | 0.972  (0.43–2.197) | 2.946  (1.223–7.098) | 0.976  (0.427–2.231) | 3.548  (1.052–11.969) | 4.525  (1.234–16.584) | 4.44  (1.377–14.319) |
| 5–7 days/week | 3.681  (1.574–8.61) | 3.796  (1.615–8.923) | 2.445  (1.043–5.73) | 1.042  (0.41–2.646) | 5.622  (1.94–16.292) | 2.182  (0.847–5.619) | 2.523  (0.463–13.737) | 2.693  (0.358–20.233) | 4.725  (0.877–25.459) |
| **Time** |  |  |  |  |  |  |  |  |  |
| <1 h/day | 1.000 (reference) | 1.000 (reference) | 1.000 (reference) | 1.000 (reference) | 1.000 (reference) | 1.000 (reference) | 1.000 (reference) | 1.000 (reference) | 1.000 (reference) |
| ≥1 h/day | 1.222  (0.767–1.947) | 1.181  (0.742–1.879) | 0.922  (0.573–1.483) | 1.374  (0.82–2.305) | 1.239  (0.722–2.129) | 0.967  (0.576–1.622) | 1.929  (0.885–4.206) | 1.104  (0.482–2.527) | 0.839  (0.38–1.851) |
| **Length** |  |  |  |  |  |  |  |  |  |
| Short-form | 1.000 (reference) | 1.000 (reference) | 1.000 (reference) | 1.000 (reference) | 1.000 (reference) | 1.000 (reference) | 1.000 (reference) | 1.000 (reference) | 1.000 (reference) |
| Long-form | 0.930  (0.543–1.595) | 0.852  (0.504–1.441) | 0.678  (0.403–1.139) | 1.371  (0.747–2.513) | 0.848  (0.453–1.588) | 1.327  (0.729–2.416) | 0.652  (0.26–1.632) | 1.509  (0.613–3.714) | 1.101  (0.449–2.699) |
| Both | 1.040  (0.599–1.807) | 1.093  (0.633–1.885) | 1.639  (0.967–2.778) | 1.161  (0.62–2.175) | 2.838  (1.474–5.468) | 1.902  (1.023–3.537) | 0.773  (0.274–2.181) | 6.815  (2.327–19.959) | 2.799  (1.02–7.683) |
| **Number** |  |  |  |  |  |  |  |  |  |
| 1 | 1.000 (reference) | 1.000 (reference) | 1.000 (reference) | 1.000 (reference) | 1.000 (reference) | 1.000 (reference) | 1.000 (reference) | 1.000 (reference) | 1.000 (reference) |
| 2 | 0.817  (0.466–1.433) | 0.785  (0.455–1.356) | 0.886  (0.518–1.514) | 1.32  (0.701–2.486) | 0.891  (0.458–1.734) | 0.771  (0.407–1.461) | 1.399  (0.618–3.17) | 1.791  (0.789–4.064) | 0.596  (0.26–1.366) |
| 3 or more | 1.606  (0.916–2.817) | 1.563  (0.9–2.716) | 0.886  (0.5–1.569) | 1.414  (0.736–2.715) | 1.07  (0.539–2.124) | 0.949  (0.493–1.829) | 1.173  (0.379–3.626) | 1.526  (0.465–5.007) | 0.834  (0.285–2.437) |
| **Influence on dietary behaviors** |  |  |  |  |  |  |  |  |  |
| No | 1.000 (reference) | 1.000 (reference) | 1.000 (reference) | 1.000 (reference) | 1.000 (reference) | 1.000 (reference) | 1.000 (reference) | 1.000 (reference) | 1.000 (reference) |
| Positive | 1.08  (0.628–1.858) | 1.325  (0.778–2.254) | 1.052  (0.619–1.788) | 0.943  (0.505–1.76) | 0.943  (0.489–1.819) | 0.777  (0.42–1.439) | 1.978  (0.689–5.678) | 2.16  (0.716–6.515) | 1.211  (0.444–3.307) |
| Negative | 1.139  (0.693–1.871) | 0.905  (0.555–1.474) | 0.771  (0.476–1.248) | 1.526  (0.546–4.265) | 0.846  (0.287–2.492) | 0.614  (0.214–1.758) | 1.139  (0.516–2.515) | 1.405  (0.631–3.13) | 0.741  (0.336–1.632) |

Values are expressed as odds ratios (95% confidence intervals).

Odds ratios were adjusted for age, sex, educational level, monthly household income, household type, alcohol consumption, physical activity, weight control attempts, health concern level, and obesity.
